# Zen and the psychological significance of meditation as related to believing

**DOI:** 10.3389/fpsyg.2022.1033021

**Published:** 2022-11-02

**Authors:** Li-Jie Du

**Affiliations:** Department of Sociology, College of Philosophy, Law, and Politics, Shanghai Normal University, Shanghai, China

**Keywords:** sitting meditation, Zen Buddhism, psychological significance, mental health, Rinzai Zen, believing

## Introduction

The word “Zen” in Buddhism is derived from the Sanskrit word “Dhyāna,“meaning” “abandoning evil” and “meditation.” It comes from Ancient Indian philosophy. This method of “sitting in silence with Pranayama and meditating to practice nyujo (Buddhist Sanskrit, Chinese: Ruding, 入定)” was introduced into Buddhism as a practice to suppress one's desires, introspect upon one's actions and problems, and keep one's heart from any evil from in the outside world. This practice is called “sitting meditation” or “meditation” in Buddhism, and through introspection (cognitively) and meditation (behaviorally), one can free oneself from external influences and make the mind peaceful. Rinzai (Lin-ji) Zen is a widely known and important school of Buddhism that advocates the practice of meditation. In modern society, there are many kinds of meditation. Some of them are related to Zen Buddhism. Others seem to be unrelated to it. This article explores Zen and the psychological significance of meditation as related to believing. It focuses on the meditation related to Zen Buddhism. However, there are other forms of meditation as well, because not all practitioners are Buddhists, although they must have beliefs about meditation for it to be effective and significant for them.

## Subsections relevant to the subject: Zen and meditation

### The origin and spread of Zen

Zen Buddhism, which advocates the practice of Zen meditation, is an essential school of Buddhism. Zen Buddhism was founded by the first Bodhidharma, who came to China from India, to the sixth master Huineng 慧能, who developed five schools and seven sects. It became the primary school of Chinese Buddhism after the Mid and Late Tang Dynasty. But after the Southern Song Dynasty, only two schools (Rinzai and Caodong 曹洞) were prevalent and brought to Japan, while the rest were not transmitted to subsequent generations. As an important school of Japanese Zen, Rinzai Zen originated mainly from the Lin-ji branch of Chinese Zen Buddhism (Chinese: Lin-ji zong, 临济宗). This meant that a disciple's satori could be directly imparted and received through the teacher's word. Some religious scholars reiterate the well-worn truism that the scholar has no direct access to the inner experience of the mystic but only access to a verbal or written account of this experience. But this claim relies on a linguistic assumption that may not be shared by the mystics themselves (Johnson, 2017).

The history of Zen meditation went from Indian Zen to Chinese Zen, and then from Chinese Zen to Japanese Zen, which then evolved into a step-by-step development of Zen from the West to the East. Just as there is no such thing as “religion” in the singular, but there are many things called “religions” based on the myriad variations in which beliefs are acquired and evolve and incorporated into one's life, so also there is no “meditation” in the singular but there are a variety of “meditations,” both religious and non-religious, that both manifest and modify beliefs. They exist because they serve the needs of human spiritual and mental health. In the process of Zen's Westward journey, the renowned scholar Suzuki Daizo played a great role in the introduction and promotion of Zen Buddhism in Europe and America. He once said, “Zen is essentially the art of gaining insight into the nature of human life, which points the way from slavery to freedom” (Erich et al., 1987). Some meditation practices are related to Buddhism and some are not. This distinction will be discussed next.

### The psychological significance of meditation related to Buddhist beliefs

During modern times, “sitting meditation” became a cultural symbol of traditional eastern psychotherapy after Zen Buddhism was introduced to the Western world by Japanese scholars. Meanwhile, in the Asian regions, such as in some cities of China, where psychology is in the process of being established as a new and developing discipline, there are a number of psychologists who draw on traditional Buddhist theories to apply them to their clinical practice in order to promote mental health. Because of the reciprocal benefit between various forms of meditation and the discipline of psychology in recent decades, the ancient Buddhism has had to adapt itself to meet the needs of modern people for spiritual health in the face of transformations in modern society. Modern Buddhist priests explain and practice Buddhist meditation from the perspective of psychology and mental health believing that it helps people relieve the pain of psychological and mental illnesses. There are some courses on the psychological bases of meditation for monks or nuns in some Buddhist colleges and institutes in Taiwan, so that they could provide psychological counseling services for their society after training. Based on their own experience in meditation, some senior monks have been able to help people become happier physically and mentally and to rid themselves of worries and fatigue by sitting in meditation and teaching sutras to the faithful (Chen and Deng, 2001).

Sitting meditation requires both “inward introspection” (which includes one's self-examination of not only one's own beliefs, but also of one's own believing process), freed from outside interference to achieve enlightenment, and adjustment of the body and mind through “meditation” to achieve a healthy state. The practice was developed in Japan into “Inner Vision Therapy,” which was absorbed by Europe more than 1,000 years later. In Germany, Hewels created the “Self-Therapy”—a psychotherapy based on sitting in meditation that had an influence on psychological and other helping techniques around the world. Coleman suggests that Buddhism's doctrine of no-self can help solve the fragmentation of self that can occur in postmodern societies (Coleman, 2001). Buddhist meditation practices underpin some cognitive behavioral therapies (which involve working with a client to change his or her negative beliefs about self) currently used in psychology to treat mental disorders (Carmody et al., 2008).

The Swiss psychoanalyst Jung concluded that “sitting meditation” has an important role in psychology. Since he wrote, some psychologists studied “meditation” and summarized the five techniques of “sitting in meditation,” which are equivalent to the “five dharma-paryaya” (dharma-gate, Chinese: Famen, 法门) in Buddhism: (a) Pranayama (to regulate the breath, Chinese: Tiaoxi, 调息); (b) Asubha (to reflections on repulsiveness, Chinese: Bujing, 不净); (c) Karunā (compassion or mercy, Chinese: Cibei, 慈悲); (d) Hetu-pratyaya (Karma, Chinese: Yinyuan, 因缘). For all the above, the practitioner must use reason and calmness to understand the cause and effect of things and use reason to overcome evil thoughts. Finally, (e) Patha (chanting, Chinese: Songnian, 诵念), which means imagining the Buddha's sitting posture and chanting his name to get rid of fear, conquer desires, and reach a state of purity and peace. This method is also used in suggestion therapy, such as with self-referral words, imagination, etc., (Xu, 2007). Success in all five of the above cases hinges on believing being central to the process of meditation; and in this way it demonstrates its significance.

### The psychological significance of meditation in non-religious believing

In recent years, sitting meditation has become a fashion in some regions, but not necessarily practiced as part of one's religiousness. The psychological and mental health fields have introduced parts of oriental Zen theory into their own disciplinary areas and developed a body of knowledge and discourse that has been de-contextualized over a long period of time so that it longer puts an emphasis on having originated from Rinzai Zen Buddhism. For example, Mindfulness meditation, in particular, has often been as part of psychotherapy with little or no attention to its religious roots or underpinnings. Current teaching and practice of mindfulness are limited to focus on one's personal wellbeing, which has therapeutic benefits but is only a small part of the wisdom, philosophy, and fundamental principles of Buddhist teaching and practice. The use of mindfulness in such a limited fashion has caused concern and raised questions. Nonetheless, mindfulness is more than that which meets the breath. It has a more powerful message and a deeper purpose; it is fundamentally about the human condition and liberation (Manikam, 2016).

Modern people are more likely to use sitting meditation as an exercise related to the promotion of spiritual and mental health. Shanghai Yufo Temple 上海玉佛寺 in China has held a free 2-day sitting meditation activity for many years, and participants do not need to have Buddhist beliefs, nor do they need to pay any fees, which are open to the public and very popular among urban young people (Du, 2015). Further research is needed to understand the implications of isolating such practices from their historical roots and religious contexts.

Many modern psychologists have highly evaluated the psychological significance of “sitting meditation” and generally believe that “sitting meditation” is not only a mystical religious system but also a psychological practice in the modern sense. Van Gordon et al. (2017) mentioned that a number of studies had investigated the utility of a secular (but Buddhist based) 8-week intervention known as meditation awareness training (MAT), which assigns to training participants in the concept and practice of emptiness (as well as other Buddhist meditative and spiritual techniques). Findings—including from clinical case studies as well as randomized and non-randomized controlled trials—have shown that MAT can improve: (a) work-related stress, (b) stress, anxiety, and depression, (c) workaholism, (d) co-occurring schizophrenia and pathological gambling, and (e) job satisfaction, organizational citizenship, and job performance (Shonin and Van Gordon, 2015).

By what processes does meditation have these effects? Such effects are related to what someone comes to believe and how they come to believe it. Meditation could also be related to the processes through which someone's beliefs change over time—for example, from pre-to-post meditation. It is exactly such hoped-for changes that many people practice mindfulness meditation. And if the changes that they want happen, they come to “believe in” meditation more than they did before—which illustrates how the believing process is not static, but fluid. This illustrates a different kind of significance of mediation, i.e., that practicing meditation involves processes of believing at its core.

### Other significance of meditation related to the believing

Scholars have increasingly become interested in silence and its role in social interaction during Buddhist meditation (Kurzon, 2007, 2011; Ephratt, 2008). Fennell (2012) analyzed the silences created in an unprogrammed Quaker Meeting for Worship as well as the meditation practices of three Buddhist groups: a sitting group associated with a Vipassana organization, a Zen sitting group under the leadership of a person ordained by Thich Nhat Hanh, and a temple under the leadership of a Zen priest of the Soto school. Such participants were able to get something out of sitting with the groups because the leaders treated learning as a shared experience. They were willing to listen to and learn with other participants, and created spaces for participants to share their perspectives. Some researchers consider silence in a spiritual context as fostering intrapersonal communication (Smith et al., 2010). Such silence operates as a conduit for people to communicate with God, deities, nature, or the “self” (Bauman, 1993; Jaworski, 1993; Dandelion, 1996; Lightstone et al., 2006; Levine, 2008), but not necessarily other people. Quakers feel they can communicate with God (Fox, 1671), and Buddhism “has in some cases been reconfigured as a technique for self-discovery” (McMahan, 2008). Even when silence is seen as active, it may be construed as functioning to turn people inward and away from each other. Instead, meditation demonstrates how silence for these groups is not necessarily socially isolating. For instance, participants not only learned, practiced, and reflected on silence together, but drew connections between the practice of silence and changes in everyday social interactions, including bringing compassion into them (Fennell, 2012).

## Discussion and conclusion

Meditation has its roots in Zen Buddhism, but later developed in a number of different variations. This article explores “sitting meditation,” not only as a mystical religious practice but also as a spiritual practice related to the believing processes understood in modern psychology. Buddhism is full of educational psychology theories, and the Buddha himself was an effective educator. The Buddha made meticulous observations of the human mind, revealed the incredible potential of the human heart, and proposed a series of operational techniques for self-knowledge, self-purification of the mind, and development of the potential of the mind (Chen, 2006). Besides those related to Buddhism, there are other types of meditation whose practitioners engage in believing in order for meditation to be effective and meaningful to them. They may not be Buddhist believers, but believing certain things is an intimate component in the very process of performing the practice of meditation, for the practice can be significant for them only if it is underpinned by believing. The dialogue between believing and psychology makes it possible to tackle more issues to be addressed in the fields of religious spirituality and mental health.

## Author contributions

The author confirms being the sole contributor of this work and has approved it for publication.

## Funding

This article was supported by the National Social Science Fund of China under the project “A study of the transmission patterns and development paths of Chinese culture among new Chinese immigrants in Canada” (Project No. 22BMZ141) and the National Social Science Fund of China under the major project “Research on the discourse system of religious sociology with Chinese characteristics and its local knowledge structure” (Project No. 18ZDA230).

## Conflict of interest

The author declares that the research was conducted in the absence of any commercial or financial relationships that could be construed as a potential conflict of interest.

## Publisher's note

All claims expressed in this article are solely those of the authors and do not necessarily represent those of their affiliated organizations, or those of the publisher, the editors and the reviewers. Any product that may be evaluated in this article, or claim that may be made by its manufacturer, is not guaranteed or endorsed by the publisher.
